# Supplementary material for: HIVprotI: an integrated web based platform for prediction and design of HIV proteins inhibitors
Source: J Cheminform. 2018 Mar 9;10:12. doi: 10.1186/s13321-018-0266-y (PMC5845081; doi:10.1186/s13321-018-0266-y)
Supplement: Supplementary file 2 — Additional file 2. Source code of HIVProtI web server. [file 13321_2018_266_MOESM2_ESM.zip › HIVprotI_Source-code/hivprotI/weka-3-6-6/doc/weka/gui/beans/IncrementalClassifierEvent.html]

IncrementalClassifierEvent


---


|  |  |  |
| --- | --- | --- |
| **Overview** \| **Package** \| **Class** \| **Tree** \| **Deprecated** \| **Index** \| **Help** \| **Weka's home** | |  |
| **PREV CLASS**   **NEXT CLASS** | **FRAMES**    **NO FRAMES**     **All Classes** |  |
| SUMMARY: NESTED \| FIELD \| CONSTR \| METHOD | DETAIL: FIELD \| CONSTR \| METHOD |  |


---


## weka.gui.beans Class IncrementalClassifierEvent

```
java.lang.Object
  java.util.EventObject
      weka.gui.beans.IncrementalClassifierEvent
```

**All Implemented Interfaces:**: java.io.Serializable

---

``` public class IncrementalClassifierEvent extends java.util.EventObject ```

Class encapsulating an incrementally built classifier and current instance

**Since:**
:   1.0

**Version:**
:   $Revision: 1.9 $

**Author:**
:   Mark Hall

**See Also:**: `EventObject`, Serialized Form

---

| **Field Summary** | |
| --- | --- |
| `static int` | `BATCH_FINISHED` |
| `static int` | `NEW_BATCH` |
| `static int` | `WITHIN_BATCH` |


**Constructor Summary**

`IncrementalClassifierEvent(java.lang.Object source)`

`IncrementalClassifierEvent(java.lang.Object source,
Classifier scheme,
Instance currentI,
int status)`
  
          Creates a new `IncrementalClassifierEvent` instance.

`IncrementalClassifierEvent(java.lang.Object source,
Classifier scheme,
Instances structure)`
  
          Creates a new incremental classifier event that encapsulates
header information and classifier.


| **Method Summary** | |
| --- | --- |
| `Classifier` | `getClassifier()`             Get the classifier |
| `Instance` | `getCurrentInstance()`             Get the current instance |
| `int` | `getStatus()`             Get the status |
| `Instances` | `getStructure()`             Get the instances structure (may be null if this is not a NEW\_BATCH event) |
| `void` | `setClassifier(Classifier c)` |
| `void` | `setCurrentInstance(Instance i)`             Set the current instance for this event |
| `void` | `setStatus(int s)`             Set the status |
| `void` | `setStructure(Instances structure)`             Set the instances structure |

**Methods inherited from class java.util.EventObject**

`getSource, toString`

**Methods inherited from class java.lang.Object**

`equals, getClass, hashCode, notify, notifyAll, wait, wait, wait`

**Field Detail**

### NEW\_BATCH

```
public static final int NEW_BATCH
```

**See Also:**: Constant Field Values

---


### WITHIN\_BATCH

```
public static final int WITHIN_BATCH
```

**See Also:**: Constant Field Values

---


### BATCH\_FINISHED

```
public static final int BATCH_FINISHED
```

**See Also:**: Constant Field Values


**Constructor Detail**

### IncrementalClassifierEvent

```
public IncrementalClassifierEvent(java.lang.Object source,
                                  Classifier scheme,
                                  Instance currentI,
                                  int status)
```

:   Creates a new `IncrementalClassifierEvent` instance.

    **Parameters:**: `source` - the source of the event: `scheme` - the classifier: `currentI` - the current instance: `status` - the status

---


### IncrementalClassifierEvent

```
public IncrementalClassifierEvent(java.lang.Object source,
                                  Classifier scheme,
                                  Instances structure)
```

:   Creates a new incremental classifier event that encapsulates
    header information and classifier.

    **Parameters:**: `source` - an `Object` value: `scheme` - a `Classifier` value: `structure` - an `Instances` value

---


### IncrementalClassifierEvent

```
public IncrementalClassifierEvent(java.lang.Object source)
```


**Method Detail**

### getClassifier

```
public Classifier getClassifier()
```

:   Get the classifier

    :   **Returns:**: the classifier

---


### setClassifier

```
public void setClassifier(Classifier c)
```

---


### getCurrentInstance

```
public Instance getCurrentInstance()
```

:   Get the current instance

    :   **Returns:**: the current instance

---


### setCurrentInstance

```
public void setCurrentInstance(Instance i)
```

:   Set the current instance for this event

    :   **Parameters:**: `i` - an `Instance` value

---


### getStatus

```
public int getStatus()
```

:   Get the status

    :   **Returns:**: an `int` value

---


### setStatus

```
public void setStatus(int s)
```

:   Set the status

    :   **Parameters:**: `s` - an `int` value

---


### setStructure

```
public void setStructure(Instances structure)
```

:   Set the instances structure

    :   **Parameters:**: `structure` - an `Instances` value

---


### getStructure

```
public Instances getStructure()
```

:   Get the instances structure (may be null if this is not
    a NEW\_BATCH event)

    :   **Returns:**: an `Instances` value


---


|  |  |  |
| --- | --- | --- |
| **Overview** \| **Package** \| **Class** \| **Tree** \| **Deprecated** \| **Index** \| **Help** \| **Weka's home** | |  |
| **PREV CLASS**   **NEXT CLASS** | **FRAMES**    **NO FRAMES**     **All Classes** |  |
| SUMMARY: NESTED \| FIELD \| CONSTR \| METHOD | DETAIL: FIELD \| CONSTR \| METHOD |  |


---
